# Supplementary material for: Weakening of the cognition and height association from 1957 to 2018: Findings from four British birth cohort studies
Source: eLife. 2023 Apr 6;12:e81099. doi: 10.7554/eLife.81099 (PMC10079289; doi:10.7554/eLife.81099)
Supplement: Supplementary file 1. — (a) Cognitive measures in four British birth cohort studies used in subsequent cross-cohort analysis: procedures and scoring, with links provided to further information. (b) The numbers of unique scores in each cognition test ) The numbers of unique scores in each cognition test. (c) Descriptive statistics, observed and imputed data. Imputed data drawn from 32 imputations. [file elife-81099-supp1.docx]

**Supplementary file for:**

**Weakening of the cognition and height association from 1957 to 2018: findings from four British birth cohort studies**

David Bann, Liam Wright, Neil M Davies, Vanessa Moulton

**Supplementary file 1a. Cognitive measures in four British birth cohort studies used in subsequent cross-cohort analysis: procedures and scoring, with links provided to further information.**

|  | 1946c | 1958c | 1970c | 2001c |
| --- | --- | --- | --- | --- |
| Age 10/11 years: Verbal reasoning | General Ability Test, [link](https://www.closer.ac.uk/cross-study-data-guides/cognitive-measures-guide/nshd-cognition/nshd-age-11-general-ability-test/)  Verbal ability subscale  This test consisted of 40 verbal items (a subset of 80 multiple choice questions including both verbal and non-verbal items).  Before the test was administered the child was shown four examples which the child and teacher completed together.  For the verbal items the child was presented with an example set of four words that were linked either logically, semantically or phonologically;  and were asked to select the correct word (out of 5 options) to complete the series.  Duration: 30 minutes for 80 items  Each correct answer given 1 mark and 0 for incorrect answer. Total score ranges from 0 to 40. | General Ability Test, [link](https://www.closer.ac.uk/cross-study-data-guides/cognitive-measures-guide/ncds-cognition/ncds-age-11-general-ability-test-verbal-and/)  Verbal ability subscale  The test consisted of 40 verbal items (a subset of 80 multiple choice questions including both verbal and non-verbal items). Before the test was administered the child was shown four examples which the child and teacher completed together. For the verbal items the child was presented with an example set of four words that were linked either logically, semantically or phonologically Next to the examples were three words, from the list, the child was required to underline the missing item which completed the sequence. Duration: 30 minutes for 80 items  Each correct answer given 1 mark and 0 for incorrect answer. Total score ranges from 0 to 40. | BAS Word Similarities, [link](https://www.closer.ac.uk/cross-study-data-guides/cognitive-measures-guide/bcs70-cognition/bcs70-age-10-bas-similarities-bas/)  The test consisted of 21 items made up of 3 words e.g. orange, banana, strawberry. The teacher read the three words and asked the child to name another word consistent with the group i.e. another type of fruit. The child then had to say what the words had in common i.e. they are all fruits. When the child was unable to name a group example and name on four successive attempts the test was stopped. Duration: Total 30 minutes for 4 BAS tests  1 point for every group example and group name, but no points if only one was correct, giving a maximum score of 21.  In this study only the group name scores were used, to aid comparability with the verbal reasoning tests administered in the other cohorts. | BAS II Verbal Similarities, [link](https://www.closer.ac.uk/cross-study-data-guides/cognitive-measures-guide/mcs-cognition/mcs-age-11-bas-ii-verbal-similarities/)  Verbal Similarities was modified to be administered with the help of the CAPI programme. The general rule in BAS assessments is that the older the child the further into the assessment they start. As all of the cohort children were approximately the same age, they all started the assessment in the same place, at item 16, after completing Example A. The child was given three stimulus words and asked to name the class to which all the examples belong.  All items (except Example A) are scored 1 or 0 points  Start at item 16 (age relevant start point) to item 28. The tests will terminate at this point (item 28) unless:  There are less than 3 incorrect responses (the test continues from item 29 to 33) or if less than 3 correct answers (the test goes back to item 8 to item 15)  There are 5 consecutive incorrect answers and less than 3 correct, the assessment will stop and go back to items 8 to 15. If items 8 to 16 are difficult the test is routed back to item 1 |

| 14/16 years: Reading/vocabulary | The Watts-Vernon Reading Test, [link](https://www.closer.ac.uk/cross-study-data-guides/cognitive-measures-guide/nshd-cognition/nshd-age-15-wattsvernon-reading-test/)  Participants were presented with a list of 35 sentences, and were asked to underline the correct word (from 5 different options) to complete each sentence, e.g. "You can buy stamps at a post (station, house, shop, man, office)". The total time of administration was approximately 15 minutes (10 minutes working time).  One mark for each correct sentence (0 - 35) | Reading Comprehension Test, [link](https://www.closer.ac.uk/cross-study-data-guides/cognitive-measures-guide/ncds-cognition/ncds-age-16-reading-comprehension-test/)  The test consisted of 35 sentences. Before the test was administered the child was shown two examples which the child and teacher completed together. The child was required to read a sentence and choose from a selection of 5 words the most appropriate to complete the sentence. From the list, the child was required to underline the missing item which completed the sentence. The test was conducted under timed conditions and within time-limit.  One mark for each correct sentence (0 - 35) | APU Vocabulary Test, [link](https://www.closer.ac.uk/cross-study-data-guides/cognitive-measures-guide/bcs70-cognition/bcs70-age-16-apu-vocabulary-test-applied-psychology/)  75 words in the test. Each word was followed by a multiple-choice list of 5 words from which the respondent picked the one with the same meaning as the first word. The test got progressively harder.  75 items; 1 point for each correct response | APU Vocabulary Test, [link](https://www.closer.ac.uk/cross-study-data-guides/cognitive-measures-guide/mcs-cognition/mcs-age-14-apu-vocabulary-test-applied-psychology/)  20 words in the test. Each word was followed by a multiple-choice list of 5 words from which the respondent picked the word with the same meaning as the original word. The test got progressively harder. In addition, the task was timed, 4 minutes was allowed and a warning was displayed on screen with one minute remaining and countdown from 60 seconds was shown.  20 items; 1 point for each correct response, 0 for incorrect or not attempted |
| --- | --- | --- | --- | --- |

| 10/11 years: Maths  (note: assessed at 7 years in 2001c) | Arithmetic Test, [link](https://www.closer.ac.uk/cross-study-data-guides/cognitive-measures-guide/nshd-cognition/nshd-age-11-arithmetic-test/)  This test consisted of 50 questions (20 mechanical sums, 30 problem questions). Questions assessed ability to add, subtract, multiply and divide. The overall testing session at age 11 lasted under 2 hours.  One mark for each solved problem (0 - 50) | Mathematics Test, [link](https://www.closer.ac.uk/cross-study-data-guides/cognitive-measures-guide/ncds-cognition/ncds-age-11-mathematics-test-2/)  The test consisted of 40 items. The test included, number skills, fractions, measures and geometry. Most questions were calculated directly, with a few involving multiple-choice answers.  One mark was awarded for each correct answer | Friendly Maths Test, [link](https://www.closer.ac.uk/cross-study-data-guides/cognitive-measures-guide/bcs70-cognition/bcs70-age-10-friendly-maths-test/)  The test consisted of a total of 72 multiple choice questions. Within each of the areas covered, the questions increased in difficulty. The test was stopped if the child failed six consecutive items.  72 items (Basic arithmetic skills (36 items), measures (16 items), algebra (6 items), geometry (10 items) and statistics (4 items) | NFER Progress in Maths (adapted), [link](https://www.closer.ac.uk/cross-study-data-guides/cognitive-measures-guide/mcs-cognition/mcs-age-7-nfer-progress-in-maths-adapted/)  The test was adapted from the NFER Progress in Maths test (Cres Fernandes, NFER). All CM's had to complete an initial test and based on their score they were routed to an easier, medium or harder section. An item response scaling method (Rasch) was used to scale the results of the easy, medium and hard subtest scores to the equivalent original raw scores.  1 - 7 completed by all; routed into easier, medium and harder. Items 2 to 4, 13 and 17 score 2 points, all other items score 1 point |
| --- | --- | --- | --- | --- |

| 15/17 years: Maths | Mathematics Test, [link](https://www.closer.ac.uk/cross-study-data-guides/cognitive-measures-guide/nshd-cognition/nshd-age-15-mathematics/)  Participants were administered a 47-item mathematics test, which tested arithmetic, geometry, trigonometry, and algebra.  Duration: The total time of administration was approximately 30 minutes (25 minutes working time). Data were heavily positively skewed, indicating the test was too difficult.  One mark for each correct item (0 - 47) | Mathematics Test, [link](https://www.closer.ac.uk/cross-study-data-guides/cognitive-measures-guide/ncds-cognition/ncds-age-16-mathematics-test/)  The test consisted of 31 items. The test included, number skills and geometry using 27 multiple-choice and 4 true or false questions.  Duration: The test was conducted under timed conditions and within time-limit (45 minutes)  31 items, each correct answer received one mark. The total of possible marks for the test was 31. | APU Arithmetic Test, [link](https://www.closer.ac.uk/cross-study-data-guides/cognitive-measures-guide/bcs70-cognition/bcs70-age-16-apu-arithmetic-test-applied-psychology/)  Multiple-choice: each question had five possible answers, only one of which was correct. The test gets progressively harder, starting with simple addition, multiplication, division and subtraction questions and ending with more complex mathematical calculations and problems to solve. Duration: 30 minutes  60 items, 1 point for each correct response | Number Analogies, [link](https://cls.ucl.ac.uk/wp-content/uploads/2020/09/MCS7-user-guide-Age-17-ed1.pdf)  Number Analogies is a short version of the Quantitative Reasoning Battery which aims to assess reasoning ability with numbers. The test forms part of Granada Learning (GL)-Assessments’ Cognitive Ability Tests. Cohort members had 6 minutes to  work through the 10 multiple choice questions, with one point given per correct answer. |
| --- | --- | --- | --- | --- |

**Supplementary file 1b. The numbers of unique scores in each cognition test**

| Test | Cohort | Mean | SD | Min. Score | Max. Score | N | Observed | # Unique Scores | Range used in sensitivity analysis |
| --- | --- | --- | --- | --- | --- | --- | --- | --- | --- |
| Maths @ 7/11 | 1946c | 26.4 | 11.7 | 0 | 50 | 5,362 | 4,015 | 51 | 0-28 |
|  | 1958c | 16.8 | 10.3 | 0 | 40 | 17,109 | 13,533 | 41 | 0-28 |
|  | 1970c | 44.0 | 12.3 | 1 | 72 | 16,549 | 11,392 | 71 | 0-28 |
|  | 2001c | 18.4 | 5.8 | 0 | 28 | 13,342 | 12,068 | 21 | 0-28 |
| Maths @ 15/17 | 1946c | 14.3 | 10.2 | 0 | 46 | 5,362 | 4,003 | 47 | 0-10 |
|  | 1958c | 12.8 | 7.0 | 0 | 31 | 17,109 | 11,256 | 32 | 0-10 |
|  | 1970c | 36.8 | 11.8 | 0 | 60 | 16,549 | 3,634 | 61 | 0-10 |
|  | 2001c | 5.2 | 2.7 | 0 | 10 | 13,342 | 8,485 | 11 | 0-10 |
| Verbal @ 10/11 | 1946c | 23.4 | 9.2 | 0 | 40 | 5,362 | 4,022 | 41 | 0-21 |
|  | 1958c | 22.2 | 9.3 | 0 | 40 | 17,109 | 13,540 | 41 | 0-21 |
|  | 1970c | 12.6 | 2.5 | 0 | 21 | 16,549 | 11,235 | 22 | 0-21 |
|  | 2001c | 120.4 | 17.2 | 10 | 179 | 13,342 | 11,535 | 40 | 0-21 |
| Vocab @ 14/16 | 1946c | 24.5 | 6.6 | 0 | 35 | 5,362 | 4,002 | 36 | 0-19 |
|  | 1958c | 25.5 | 6.9 | 0 | 35 | 17,109 | 11,315 | 36 | 0-19 |
|  | 1970c | 10.2 | 4.2 | 0 | 20 | 16,549 | 5,613 | 21 | 0-19 |
|  | 2001c | 7.1 | 2.6 | 0 | 19 | 13,342 | 9,609 | 19 | 0-19 |

**Supplementary file 1c. Descriptive statistics, observed and imputed data. Imputed data drawn from 32 imputations.**

|  | | 1946c | | | 1958c | | | 1970c | | | 2001c | | |
| --- | --- | --- | --- | --- | --- | --- | --- | --- | --- | --- | --- | --- | --- |
|  | Variable | Observed | Missing % | Imputed | Observed | Missing % | Imputed | Observed | Missing % | Imputed | Observed | Missing % | Imputed |
|  | Sample Size | 5,348 |  | 5,348 | 17,490 |  | 17,490 | 17,617 |  | 17,617 | 17,057 |  | 17,057 |
| Sex | Female | 2,538 (47.46%) | 0% | 2,535.5 (47.41%) | 8,445 (48.28%) | 0% | 8,445 (48.28%) | 8,468 (48.07%) | 0% | 8,468 (48.07%) | 8,275 (48.51%) | 0% | 8,287.61 (48.59%) |
|  | Male | 2,810 (52.54%) |  | 2,812.5 (52.59%) | 9,045 (51.72%) |  | 9,045 (51.72%) | 9,149 (51.93%) |  | 9,149 (51.93%) | 8,782 (51.49%) |  | 8,769.39 (51.41%) |
|  | Height @ Age 11 (Z-Score) | -0.33 (1.04) | 26.61% | -0.41 (1.03) | -0.22 (1.02) | 30.51% | -0.25 (1.03) | -0.17 (1.01) | 30.45% | -0.18 (1.01) | 0.27 (1.03) | 31.88% | 0.29 (1) |
|  | Height @ Age 16 (Z-Score) | -0.47 (1.04) | 32.24% | -0.54 (1.03) | -0.33 (1.02) | 40.21% | -0.35 (1.02) | -0.09 (1.18) | 58.36% | -0.14 (1.16) | 0.23 (1.01) | 41.12% | 0.27 (0.98) |
|  | Maternal Height (cm) | 161.2 (6.35) | 21.35% | 160.81 (6.38) | 162.02 (6.46) | 26.23% | 161.99 (6.45) | 161.3 (6.65) | 25.58% | 161.28 (6.63) | 163.65 (7.03) | 5.83% | 164.14 (6.93) |
|  | Paternal Height (cm) | 173.4 (7.94) | 35.71% | 172.68 (8.07) | 174.53 (7.42) | 27.94% | 174.47 (7.42) | 175.24 (7.53) | 28.91% | 175.21 (7.51) | 177.85 (7.41) | 31.45% | 178.28 (7.23) |
| Father's Social Class | I Professional | 238 (6.08%) | 26.83% | 161.31 (3.02%) | 689 (5.56%) | 29.13% | 952.12 (5.44%) | 751 (6.27%) | 32.05% | 1,078.22 (6.12%) | 469 (5.17%) | 46.8% | 850.12 (4.98%) |
|  | II Intermediate | 761 (19.45%) |  | 748.82 (14%) | 2,290 (18.47%) |  | 3,182.47 (18.2%) | 2,867 (23.95%) |  | 4,160.09 (23.61%) | 3,834 (42.25%) |  | 7,354.00 (43.11%) |
|  | III Skilled Manual | 1,331 (34.01%) |  | 2,498.96 (46.73%) | 5,359 (43.23%) |  | 7,585.34 (43.37%) | 5,287 (44.17%) |  | 7,833.41 (44.47%) | 2,030 (22.37%) |  | 3,700.60 (21.7%) |
|  | III Skilled Non-Manual | 601 (15.36%) |  | 484.02 (9.05%) | 1,166 (9.41%) |  | 1,622.09 (9.27%) | 1,105 (9.23%) |  | 1,621.81 (9.21%) | 1,197 (13.19%) |  | 2,257.30 (13.23%) |
|  | IV Partly Skilled | 742 (18.96%) |  | 1,015.55 (18.99%) | 2,152 (17.36%) |  | 3,076.56 (17.59%) | 1,478 (12.35%) |  | 2,198.56 (12.48%) | 1,288 (14.19%) |  | 2,394.23 (14.04%) |
|  | V Unskilled | 240 (6.13%) |  | 439.34 (8.22%) | 740 (5.97%) |  | 1,071.41 (6.13%) | 483 (4.03%) |  | 724.91 (4.11%) | 256 (2.82%) |  | 500.75 (2.94%) |
| Mother's Education | High | 1,235 (28.54%) | 19.07% | 1,115.51 (20.86%) | 4,224 (24.93%) | 3.14% | 4,384.25 (25.07%) | 5,681 (34.44%) | 6.37% | 6,069.03 (34.45%) | 8,472 (49.86%) | 0.39% | 9,013.27 (52.84%) |
|  | Low | 3,093 (71.46%) |  | 4,232.49 (79.14%) | 12,717 (75.07%) |  | 13,105.75 (74.93%) | 10,813 (65.56%) |  | 11,547.97 (65.55%) | 8,518 (50.14%) |  | 8,043.73 (47.16%) |
